# Pressure optimization for hydraulic-electric hybrid biped robot power unit based on genetic algorithm

**DOI:** 10.1038/s41598-022-26852-1

**Published:** 2023-01-02

**Authors:** Pengyu Zhao, Anhuan Xie, Shiqiang Zhu, Lingyu Kong

**Affiliations:** 1 Zhejiang Lab, 1818 West Wenyi Road, Hangzhou, 311121 Zhejiang People’s Republic of China; 2 Zhejiang University, 38 Zheda Road, Hangzhou, 310027 Zhejiang People’s Republic of China

**Keywords:** Energy science and technology, Mechanical engineering

## Abstract

Biped robots have attracted increasing attention because of their flexible movement and strong adaptability to the surroundings. However, the small output torque and the weak impact resistance of the motor drive, as well as the large energy consumption of the hydraulic drive limit the performance of the biped robot drive system. Aiming at these shortcomings, an electric-hydraulic hybrid drive system of biped robot was proposed in this paper. The robot platform was designed based on the prototype of the Zhejiang Lab biped robot. The model of the hydraulic drive system and mechanical structure was established to analyze the dynamic characteristic and the load force during walking. The value function reflecting the energy consumption of the hydraulic drive system was proposed. The pressure of the accumulator in the hydraulic power unit was selected as the control parameter. In order to get the minimum value of the value function, so as to reduce the energy consumption of the hydraulic driving system, the control parameters were optimized by using the genetic algorithm. From the simulation results, the proposed optimization algorithm can improve efficiency by 3.49%.

## Introduction

Mobil robots can be categorized as wheeled, tracked and legged type according to the different modes of movement. Among them, legged robots have been paid increasing attention because of their flexible movement and strong adaptability to the surroundings. The driving modes of legged robots developed in recent years mainly include the motor drive and the hydraulic drive^1^.

The hydraulic drive has many excellent characteristics, making it an ideal choice for highly dynamic articulated robots. On one hand, the power density of hydraulic drives is high^2^. For example, the power density of the Shandong University SCALF-III robot^3^ which is driven by the hydraulic system, can reach 7 kW/kg, while the power density of the EC60-400 W robot^4^ and TBM(S)-12,913-B robot^5^ which are driven by the motor, is 0.17 kW/kg and 0.43 kW/kg, respectively. On the other hand, gear transmission is not necessary in a hydraulic drive system, which makes it possible to absorb large impact load^6^. Hence, the impact resistance robustness, stiffness and bandwidth are improved. However, the low energy efficiency and serious heating of the hydraulic system have become one of important factors limiting its development^7^.

The hydraulic drive system can be divided into a closed pump control system and an open valve control system according to the different control components. The closed pump control system does not need a large hydraulic tank. Thus, the weight and volume of the hydraulic power unit are reduced. Also, throttling loss is avoided, which can significantly improve the efficiency of the hydraulic system and shows obvious advantages in energy saving^8^. In a typical closed system, the oil inlet and outlet of the actuator are connected to the two oil outlets of the hydraulic pump respectively. The speed and movement direction of the actuator are controlled by adjusting the rotational speed or displacement of the hydraulic pump. For example, the direct pump-controlled system utilized in legged robots can avoid the throttling loss of the servo valves and achieve high energy efficiency^9^^,^^10^. However, since the inertia mass (or moment of inertia) of a hydraulic pump variable mechanism or the rotor of a motor is much larger than that of a valve core, the response speed of the pump control system is usually slow. The load adaptability and controllability are not as good as a valve control system. Usually, the closed pump control system has a good driving effect for the system with slow motion or low requirement of response speed and control precision. It is still difficult to be applied to the legged robot.

The open valve control system, especially the servo valve controlled hydraulic actuator, has become the main driving mode of the robot hydraulic drive system. The servo valve has a high response characteristic, which meets the requirements of a robot drive system. Also, the control algorithm is mature with a good control effect. For example, the biped robots like BBH from Bath University^11^, NWPUBR-1 from Northwestern Polytechnical University^12^, and the quadruped robots like Hydraulically-powered Quadruped (HyQ) robots from the Institute of Technology in Italy^13,14,15^, SCalf from Shandong University^16,17^ and quadruped robot from Yanshan University^18,19,20^ are all driven by servo valve controlled cylinders. However, in an open valve control system with a centralized power source, the power unit should always maintain high pressure to ensure that the system has a high response characteristic. When the output force of the actuator is small, the power unit still needs to supply oil at the highest pressure, which leads to a large throttling loss of the valve port. Then the energy efficiency of the system is reduced and the heat generated by the valve is increased.

In order to reduce the energy consumption of the hydraulic drive system, many studies have been carried out in resent years. Hydraulic pump control is an effective way to reduce energy losses, such as model predictive control (MPC)^21^. The control method can reduce energy consumption with a reliable pressure-supply ability. Other effective methods mainly focus on trajectory optimization^22,23,24^ and mechanism optimization^25^. However, the limitation includes significant leakage and throttle loss. In order to further increase energy efficiency, the difference between the demand pressure of the actuator and the supplied pressure of the power unit should be reduced to minimize the pressure drop of the control valves. A multistage supply pressure system is one of the approaches. For example, multi-pump hydraulic power unit with a valve matrix is one of the attempts^26^. This power unit can significantly increase the energy utilization rate. But it increases the weight of the hydraulic system. So, the improvement of the total energy efficiency of the robot is limited.

According to the analysis above, based on the biped robot platform proposed by Zhejiang Lab, an electric-hydraulic hybrid drive system is proposed in this paper. The drive system makes full use of the advantages of the high efficiency of motor drive and the large power density of hydraulic drive. Aiming at the shortage of large energy loss and low efficiency of the hydraulic system, two accumulators are introduced in the hydraulic power unit to supply multistage pressure. Also, the pressure of the power unit is optimized and the control strategy is put forward to further reduce energy consumption. The proposed approach avoids the use of multiple motor-pump units, and can reduce throttle loss of control valves.

## System configuration

### Mechanical construction

The prototype of the Zhejiang Lab biped robot consists of a trunk, hips, thighs, calves and feet, which are hinged between adjacent body structures. There is a degree of freedom (DOF) of turnover to control the abduction/adduction of the leg and a freedom of yaw to control the direction of the leg between the hip and the thigh. The two DOFs provide no vertical bearing capacity, which reduces the demand torque. So, they are designed to be driven by electric motors.

The hip joint, knee joint and ankle joint each have a DOF of rotation. The driving torque of the hip joint and knee joint is large. The peak value of the driving torque of the two joints exceeds the maximum output torque of the electric motor under the impact load. So, the hydraulic drive is utilized. In the underdrive gait algorithm based on the virtual constraint, the ankle joint only controls the foot to be parallel to the ground, and the required torque is small. So, it is also driven by an electric motor.

In order to reduce the moving parts moment of inertia, actuators should be centrally installed on non-moving parts such as the hip, or on the structures with small rotation radius such as the upper thigh. For this purpose, the turnover and yaw motors are fixedly attached to the trunk and hips respectively. They are mounted coaxially with the rotating shaft. The ankle is driven by an electric motor mounted on the hip through two sets of connecting links. The hip hydraulic cylinder is hinged to the hip and thigh. The knee hydraulic cylinder is hinged to the thigh and calf. The structure of the designed hybrid drive robot is shown inFig. 1.

**Figure 1:**
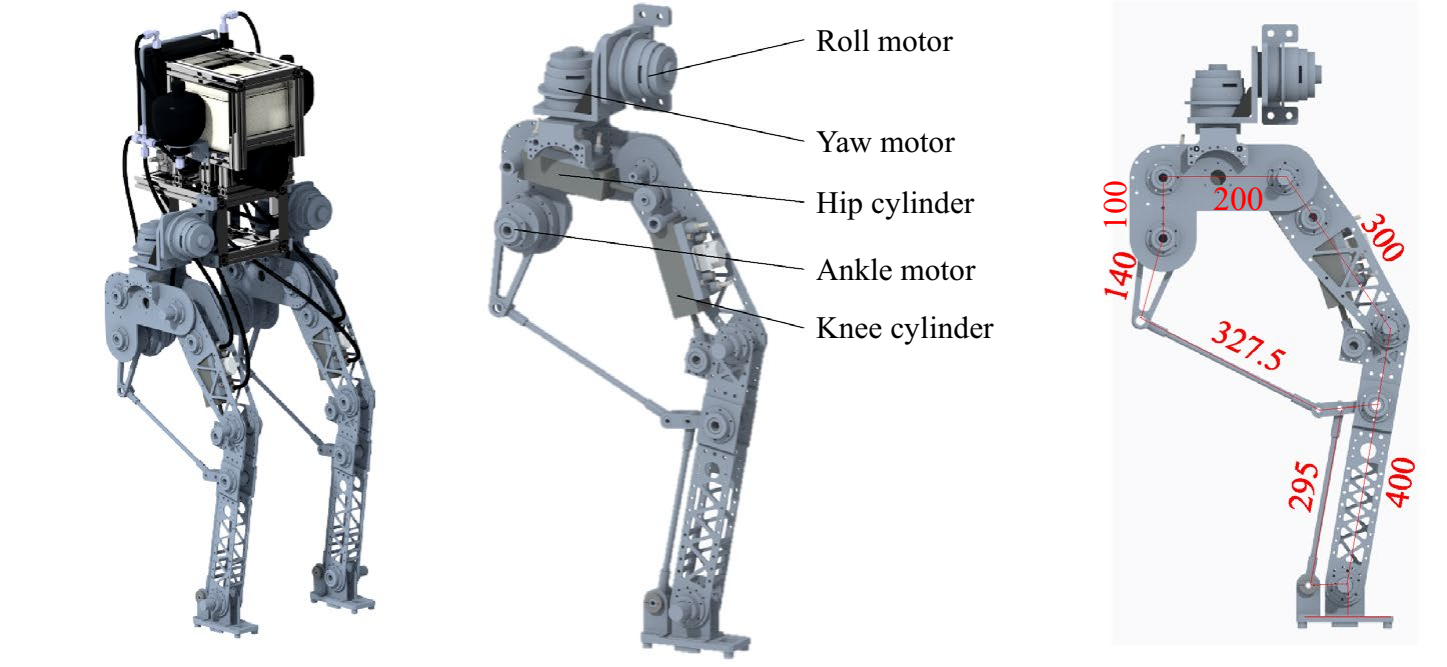
Mechanical structure of hybrid drive robot.

### Hydraulic system

In order to improve the response of the system and realize accurate position control, the servo valve controlled hydraulic cylinders are utilized as the actuators. The rated pressure of the hydraulic system is 21 MPa. Based on the calculation, the pressure of the hydraulic cylinder is generally no more than 10 MPa under normal walking conditions, and the pressure is high only under the impact load or the instantaneous rapid movement. If the outlet pressure of the pump is maintained at the rated pressure all the time, the pressure difference between the pressure port and the working ports of the servo valve is large, which increases the throttling loss of the servo valve and the input power of the hydraulic pump, resulting in a very low system efficiency. From simulation analysis, if the oil maintains 21 MPa constant pressure, the efficiency of the hydraulic system is only about 10% in the walking condition.

**Figure 2:**
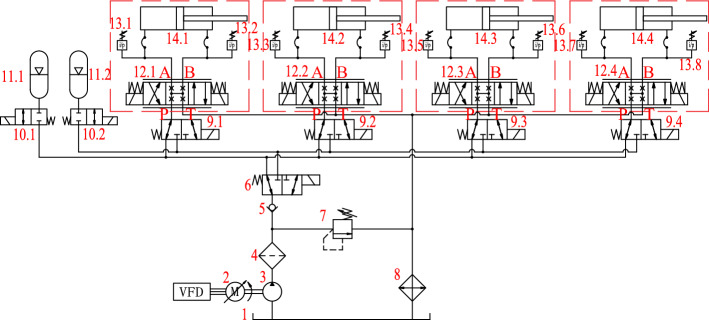
Principle of hydraulic system.

In order to improve the efficiency of the system, accumulators with high and low working pressures are used to supply oil to the system. The hydraulic pump charges the accumulator respectively according to the charging state of each accumulator. Each hydraulic cylinder accesses the high-pressure or low-pressure circuit according to the demand pressure, controlled by a directional valve. The principle of the hydraulic system is shown in Fig. 2.

(1) Oil tank, (2) Variable frequency motor, (3) Hydraulic pump, (4) Filter, (5) Check valve, (6) Two-position three-way directional control valve, (7) Relief valve, (8) Cooler, (9) Two-position three-way directional control valve, (10) Switch valve, (11) Accumulator, (12) Servo valve, (13) Pressure sensor, (14) Hydraulic cylinder.

In the system, 11.1 is a low-pressure accumulator and 11.2 is a high-pressure accumulator. The following is an example to introduce the working principle. When the pressure in the hydraulic cylinder 14.1 is lower than the set pressure of the low-pressure accumulator, the directional valve 9.1 connects the cylinder 14.1 to the low-pressure accumulator circuit. At this time, the pressure at the oil inlet P of the servo valve 12.1 is low. When the cylinder 14.1 drives the joint, the oil in the low-pressure accumulator is consumed. On the contrary, when the pressure in the cylinder 14.1 is higher than the set pressure of the low-pressure accumulator, the directional valve 9.1 connects the cylinder 14.1 to the high-pressure accumulator circuit. At this time, the pressure at the oil inlet P of the servo valve 12.1 is high pressure, and the oil in the high-pressure accumulator is consumed when the hydraulic cylinder 14.1 acts. The hydraulic pump 3 charges liquid to the low-pressure accumulator or high-pressure accumulator through the directional valve 6. In this system, the hydraulic pump charges the low-pressure accumulator most of the time. At that moment, the outlet pressure of the pump is equal to that of the low-pressure accumulator. So, the energy consumption of the hydraulic pump can be reduced.

According to the load of the robot, the parameters of the main components are calculated, as shown in Table 1.

**Table 1:** Main components parameters of hydraulic system.

Components	Parameter	Value	Components	Parameter	Value
Pump	Rated pressure/MPa	21.0	Servo valve	Rated flow rate/ L·min^−1^ (@7 MPa pressure drop)	6.9
	Displacement/mL·r^−1^	4.0		Response frequency/Hz	200
	Rotate speed/rpm	0–4000		Damping ratio	0.5
Cylinder	Piston rod diameter/mm	10	Accumulator	Volume/L	2.6
	Piston diameter/mm	25		Rated pressure/MPa	21.0
	Stroke/mm	50			

## Mathematical model

The mathematical models of servo valves, hydraulic cylinders, accumulators and pump are established. For reasons of space limitation, the specific mathematical formula is omitted. Also, the mechanical structure model is established to obtain the load force and energy consumption.

### Mechanical structure and hydraulic system

The objective of this paper is to analyze the performance of the hydraulic drive system, which drives the hip joint and knee joint. In order to simplify the simulation model, the robot movement is limited to a two-dimensional plane, which has no obvious effect on the analysis results of the hydraulic system.

**Figure 3:**
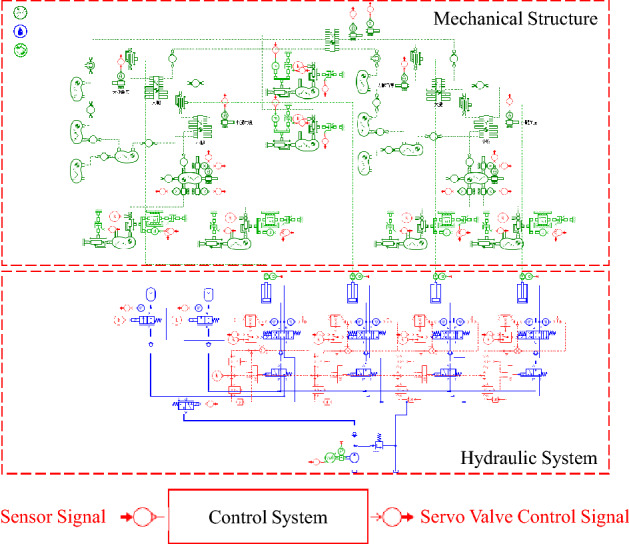
Model of mechanical structure and hydraulic system in AMESim.

**Figure 4:**
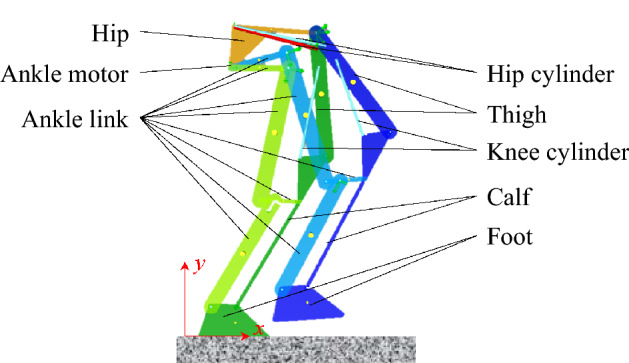
Topology model of mechanical structure.

**Figure 5:**
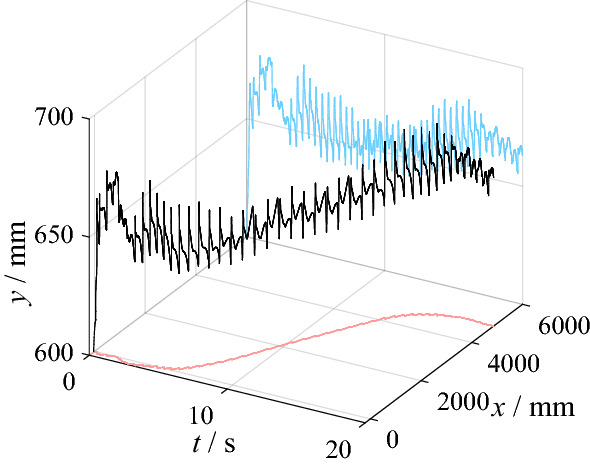
Position of robot gravity center during walking.

The physical model of the mechanical structure and the hydraulic system is established in AMESim as shown in Fig. 3. In the proposed robot, only the hip joint and knee joint are driven by hydraulic system. The two DOFs of turnover and yaw of the leg, which controls the lateral movement, has little relation with the performance of the hydraulic system. For this reason, the robot movement is limited to a two-dimensional plane, which has no obvious effect on the analysis results of the hydraulic system. The computer used to simulate the model contains an Intel Core i7-9700 K CPU. The geometric dimensioning of the mechanical structure is the same as Fig. 1, while the parameters of the main components are the same as Table 1. Figure 4 shows the topology model of the robot in the simulation. In this paper, the gait algorithm based on virtual constraint is used to control the robot walking within 20 s^27^. The gait algorithm is formulated as a large-scale programming problem. The dynamics constrains are established for the proposed robot configuration. In order to reduce the dimensionality, the system state of the hybrid dynamics model is reconstructed. Following the problem formulation and optimization, the optimized gait is applied to the biped locomotion under the design of feedback control. During the movement, the position of the robot’s gravity center is shown in Fig. 5. Figure 5 also shows the projection of the gravity center on the *x-t* plane and the *y–t* plane. The sensors in the model include angle, force and pressure sensors. The angle sensors are mounted coaxially with joints to measure the joint angles. Force sensors locate at the end of cylinder rods to measure the output force of the cylinder. Pressure sensors are installed in the cylinders and the outlet of accumulators to acquire the pressure in the hydraulic system. Based on the model, parameters such as joint angle, actuator output force and pressure can be obtained.

### Energy consumption

According to the load force, the output power of the actuator can be calculated:

1 $$ P_{{{\text{c\_out}}i}} = F_{{{\text{c}}i}} \frac{{{\text{d}}x_{{{\text{p}}i}} }}{{{\text{d}}t}} $$

where $P_{{{\text{c\_out}}}}$ is the output power of the cylinder, $F_{{\text{c}}}$ is the output force of the cylinder, $x_{{\text{p}}}$ is the displacement of the piston, $i{ = 1},{2},{3},{4}$ representing left hip, right hip, left knee and right knee respectively.

Then, the active work done by the actuator is:

2 $$ W_{{{\text{c\_out}}i}} = \int_{t = 0}^{{t_{{\text{f}}} }} {P_{{{\text{c\_out}}i}} } \cdot {\text{d}}t $$

where $W_{{{\text{c\_out}}}}$ is the active work of the cylinder, $t_{{\text{f}}}$ is the final moment of the movement. For the specific movement calculated in this paper, $t_{{\text{f}}} {\text{ = 20s}}$.

The input power of a cylinder can be expressed as:

5 $$ P_{{{\text{c\_in}}i}} = p_{{{\text{vP}}i}} q_{{{\text{vP}}i}} $$

where $P_{{{\text{c\_in}}}}$ is the input power of the cylinder, $p_{{{\text{vP}}}}$ is the pressure of servo valve port P, $q_{{{\text{vP}}}}$ is the flow rate of servo valve port P.

The input power of the hydraulic system can be expressed as:

6 $$ P_{{{\text{in}}}} { = }T_{{\varvec{p}}} \omega_{{\varvec{p}}} $$

where $P_{{{\text{in}}}}$ is the hydraulic system input power, $T_{{\varvec{p}}}$ is the pump input torque; $\omega_{{\varvec{p}}}$ is the pump rotate speed.

The energy consumed by the hydraulic system is:

7 $$ W_{{{\text{in}}}} { = }\int_{0}^{{t_{{\text{f}}} }} {P_{{{\text{in}}}} {\text{d}}t} { + }\frac{{\sum {E_{{{\text{a\_i}}}} } { - }\sum {E_{{{\text{a\_f}}}} } }}{{\eta_{{\text{a}}} }} $$

where $W_{{{\text{in}}}}$ is the hydraulic system energy consumption, $E_{{{\text{a\_i}}}}$ is the energy stored in the accumulator at the beginning of the movement; $E_{{{\text{a\_f}}}}$ is the energy stored in the accumulator at the end of the movement; $\eta_{{\text{a}}}$ is the average efficiency when the hydraulic system charges the accumulator. We take $\eta_{{\text{a}}} {\text{ = 95\% }}$.

## Power unit pressure optimization algorithm

The purpose of the proposed algorithm is to reduce the energy consumption of the hydraulic system by optimize the pressure of the power unit. The algorithm also controls the hydraulic pump to charge the accumulators to maintain the pressure within a certain range.

A value function is selected to represent the energy consumption as well as the penalty item. In order to take full use of the low-pressure accumulator, the switching time interval of the reversing valve is introduced as the penalty function item. Then the value function is:

8 $$ J_{{\text{v}}} { = }\int_{t = 0}^{{t_{{\text{f}}} }} {\left( {P_{{{\text{in}}}} + k_{{\text{p}}} \cdot \Delta t_{{\text{s}}} } \right){\text{d}}t} $$

where $\Delta t_{{\text{s}}}$ is the switching time interval; $k_{{\text{p}}}$ is the coefficient of the penalty function item:

9 $$ k_{{\text{p}}} { = }\left\{ {\begin{array}{*{20}c} {k_{{{\text{ep}}}} \left( {\frac{{\Delta t_{{{\text{set}}}} }}{{\Delta t_{{\text{s}}} }} + \frac{{\Delta t_{{\text{s}}} }}{{\Delta t_{{{\text{set}}}} }} - 2} \right)\begin{array}{*{20}c} {} & {\Delta t_{{\text{s}}} < \Delta t_{{{\text{set}}}} } \\ \end{array} } \\ {0\begin{array}{*{20}c} {} & {\Delta t_{{\text{s}}} \ge \Delta t_{{{\text{set}}}} } \\ \end{array} } \\ \end{array} } \right. $$

where $\Delta t_{{{\text{set}}}}$ is the ideal switching time interval of the reversing valve; $k_{{{\text{ep}}}}$ is the coefficient.

The pressure of the high-pressure accumulator is determined by the rated pressure of the system and the demand pressure of the actuator. There is no need to optimize the pressure of the high-pressure accumulator. Therefore, the pressure range of the high-pressure accumulator is set as 19–21 MPa. That is, the minimum working pressure of the high-pressure accumulator is $p_{{{\text{ah\_l}}}} {\text{ = 19MPa}}$ and the maximum working pressure is $p_{{{\text{ah\_h}}}} {\text{ = 21MPa}}$.

We assume that the lowest working pressure and the highest working pressure of the low-pressure accumulator are $p_{{{\text{al\_l}}}}$ and $p_{{{\text{al\_h}}}}$, respectively. In order to reduce the maximum input power of the hydraulic system, the hydraulic pump should charge the accumulators with the approximately constant flow to avoid the instantaneous large flow of oil output. Also, in order to reduce the computational complexity, external disturbances are not considered. The accumulators utilized in the system can supply the robot walk several steps without additional charging. Even though the real consumed flow by the robot changes periodically during walking, the average flow rate during the charging cycle of the accumulators can be considered approximately a constant value. Based on this situation, it is assumed that the actuators consume the oil in the high/low pressure accumulator at a constant rate of $q_{{{\text{ah\_out}}}}$ and $q_{{{\text{al\_out}}}}$, respectively. The hydraulic pump charges the high/low pressure accumulator at the flow rate of $q_{{{\text{ah\_in}}}}$ and $q_{{{\text{al\_in}}}}$. It can be obtained by equaling the consuming time to the charging time:

10 $$ \frac{{\sqrt[n]{{\frac{{p_{ah\_h} }}{{p_{ah\_l} }}}} - 1}}{{q_{ah\_out} }} = \frac{{\sqrt[n]{{\frac{{p_{al\_h} }}{{p_{al\_l} }}}} - 1}}{{q_{al\_in} - q_{al\_out} }} $$

11 $$ \frac{{\sqrt[n]{{\frac{{p_{{{\text{ah}}\_{\text{h}}}} }}{{p_{{{\text{ah}}\_{\text{l}}}} }}}} - {1}}}{{q_{{{\text{ah}}\_{\text{in}}}} - q_{{{\text{ah}}\_{\text{out}}}} }}{ = }\frac{{\sqrt[n]{{\frac{{p_{{{\text{al}}\_{\text{h}}}} }}{{p_{{{\text{al}}\_{\text{l}}}} }}}} - {1}}}{{q_{{{\text{al}}\_{\text{out}}}} }} $$

From the equations above, we can get $q_{{{\text{ah\_in}}}}$ and $q_{{{\text{al\_in}}}}$. Then the output flow rate of the pump is:

12 $$ q_{{\text{p}}} { = }k_{{\text{s}}} q_{{{\text{ah\_in}}}} { + }\left( {{1} - k_{{\text{s}}} } \right)q_{{{\text{al\_in}}}} $$

where $k_{{\text{s}}}$ is the input signal of reversing valve. When the pump charges the high-pressure accumulator, $k_{{\text{s}}} { = 1}$. Otherwise, $k_{{\text{s}}} { = 0}$.

The power unit control rule is:

1. In the initial state, the pressure of the high-pressure accumulator is the highest set working pressure $p_{{{\text{ah\_h}}}}$, and the pressure in the low-pressure accumulator is the lowest set working pressure $p_{{{\text{al\_l}}}}$.
2. When the pressure in the low-pressure accumulator drops to the lowest set pressure $p_{{{\text{al\_l}}}}$, the hydraulic pump charges the low-pressure accumulator with flow rate $q_{{{\text{al\_in}}}}$ until the pressure reaches and maintains the highest set pressure $p_{{{\text{al\_h}}}}$.
3. When the pressure in the high-pressure accumulator drops to the lowest set pressure $p_{{{\text{ah\_l}}}}$, the hydraulic pump charges the high-pressure accumulator.
4. When the pressure in the high-pressure accumulator reaches the highest set pressure $p_{{{\text{ah\_h}}}}$, the hydraulic pump is switched back to charge the low-pressure accumulator with flow rate $q_{{{\text{al\_in}}}}$. The rest can be done in the same manner.

The switching rule of the actuator access to the high/low pressure circuit is:

1. When the pressure of the two chambers of the hydraulic cylinder is lower than the minimum set pressure of the low-pressure accumulator $p_{{{\text{al\_l}}}}$, the hydraulic cylinder is connected to the low-pressure circuit.
2. When the pressure of either chamber of the hydraulic cylinder is higher than the minimum set pressure of the low-pressure accumulator $p_{{{\text{al\_l}}}}$, the hydraulic cylinder is connected to the high-pressure circuit.

The pressure range of the low-pressure accumulator is determined by the genetic algorithm. The parameters of genetic algorithm are shown in Table 2.

**Table 2:** Parameter of genetic algorithm.

Parameter	Value	Parameter	Value
Population quantity	50	Crossover probability	0.8
Scaling function	Rank	Crossover function	Scattered
Selection function	Stochastic uniform	Mutation probability	0.2
Elite count	5	Mutation interval	20

The genetic algorithm is shown below:
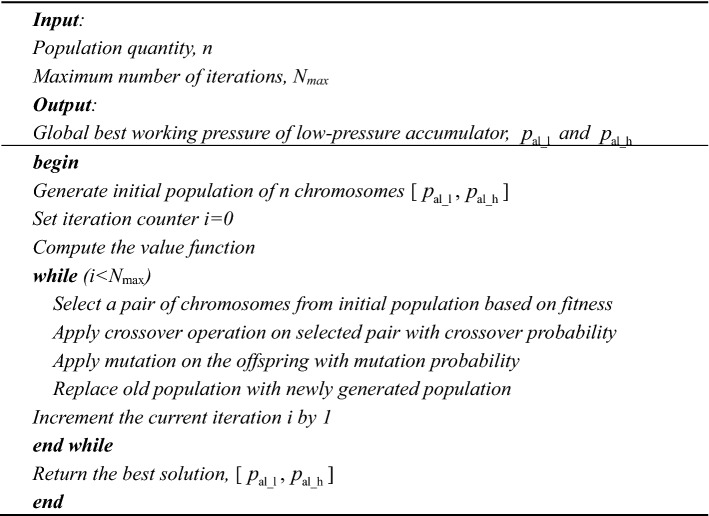


With the large-scale computation through the genetic algorithm, the parameters that make the value function obtain the global optimal value can be calculated. Then, the working pressure of the low-pressure accumulator is determined. The penalty function item in the value function ensures that the switching time interval of the reversing valve is not too short. The energy stored in the low-pressure accumulator can be fully utilized. With the determined working pressure, the hydraulic system can achieve a relatively high efficiency.

According to the pressure optimization algorithm proposed above, the data collected during robot walking include the pressure of low-pressure accumulator and high-pressure accumulator, the pressure of each chamber of the hydraulic cylinders, the rotate speed of the pump and the motion of each hydraulic cylinder. The pressure of the accumulators can be measured by the pressure sensors located at the outlet of the accumulators. The hydraulic cylinders are the ones integrated with pressure sensors which measure the pressure in the chambers. The flow that the pump supplied and the cylinders consumed are calculated by the rotate speed of the pump and the joints.

In order to compare the energy consumption, the robot walks along the gait described in Section “Mechanical Structure and Hydraulic System” in different conditions. Also, the external disturbances are ignored to reduce the complexity.

## Numerical experiment

In order to verify the effectiveness of the proposed power unit pressure optimization algorithm, the working pressures of the low-pressure accumulator calculated by the optimization algorithm are used. The energy consumption during walking is obtained. The model and parameters, as well as data acquisition process, are the same as mentioned above.

### Optimized working pressure

**Figure 6:**
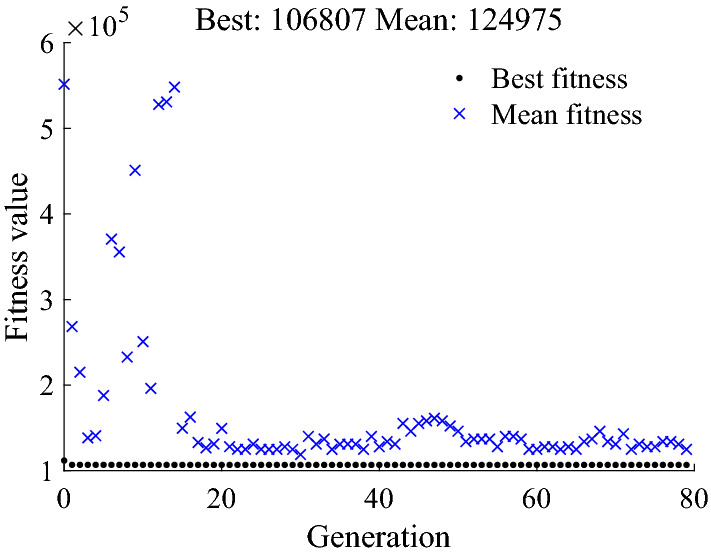
Fitness value of genetic algorithm.

Firstly, the optimized working pressure of the low-pressure accumulator is obtained according to the optimization algorithm. The mean fitness and best fitness during the training process are shown in Fig. 6.

The best solution calculated by the genetic algorithm is [$p_{{{\text{al\_l}}}}$,$p_{{{\text{al\_h}}}}$] = [6.2,8.7]. Based on the calculation result, the working pressures of the accumulators are shown in Table 3.

**Table 3:** Working pressure range of accumulators.

Accumulator	Parameter	Value/MPa
High-pressure accumulator	Highest pressure	21.0
	Lowest pressure	19.0
	Pre-charge pressure	17.1
Low-pressure accumulator	Highest pressure	8.7
	Lowest pressure	6.2
	Pre-charge pressure	5.6

**Figure 7:**
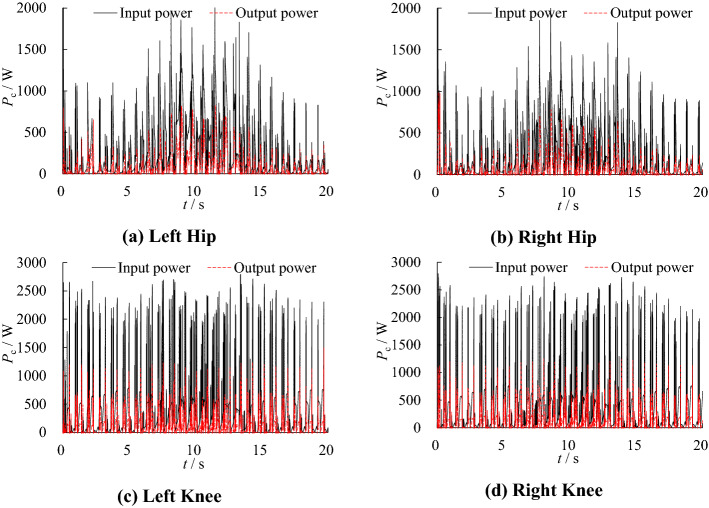
Actuator input/output power after optimization.

The simulation model described in Section “Mathematical Model” is used to simulate and analyze the robot movement. The input power and output power of each joint are shown in Fig. 7.

**Figure 8:**
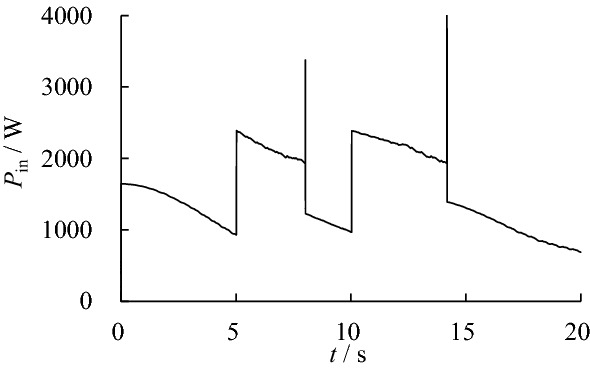
Input power of hydraulic system after optimization.

The input power of the hydraulic system after optimization is shown in Fig. 8. After optimization, the active work and energy consumption of the hydraulic system is shown in Table 4.

**Table 4:** Active work and energy consumption after optimization.

Component	Active work/J	Energy/J	Efficiency/%
Left hip cylinder	1910.27	5134.67	37.20
Right hip cylinder	1616.08	4662.51	34.66
Left knee cylinder	2538.17	9139.83	27.77
Right knee cylinder	2494.23	8850.20	28.18
Hydraulic system	8558.75	29,650.77	28.87

### Pressure without optimization

In order to calculate the power and energy consumed by the hydraulic system and provide the comparison for the power unit pressure optimization algorithm, the power unit pressure without optimization control is obtained in this section.

**Figure 9:**
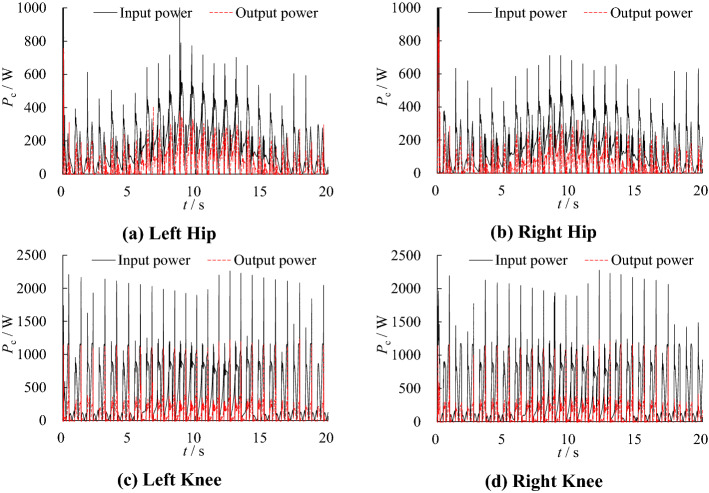
Actuator input/output power without optimization.

**Figure 10:**
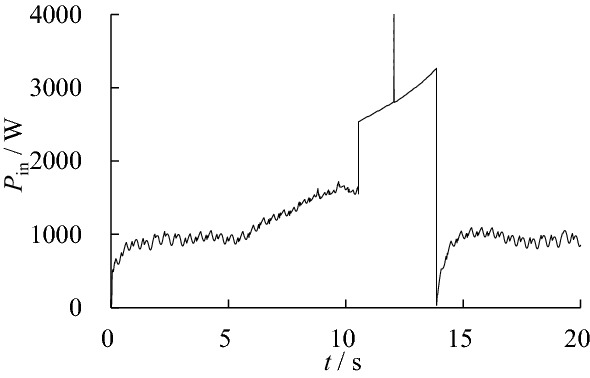
Input power without optimization.

Since the demand pressure of the hydraulic actuator is less than 10 MPa in most of the time, the pressure range of the low-pressure accumulator is set as 10–12 MPa and the high-pressure accumulator is set as 19–21 MPa. In this working condition, the input power and output power of each joint are shown in Fig. 9, the input power of the hydraulic system is shown in Fig. 10.

Based on the calculation above, the active work and energy consumption of the hydraulic system can be obtained, as shown in Table 5.

**Table 5:** Active work and energy consumption without optimization.

Component	Active work/J	Energy/J	Efficiency/%
Left hip cylinder	1470.15	3672.94	40.03
Right hip cylinder	1325.02	3488.89	37.98
Left knee cylinder	2515.99	9764.15	25.77
Right knee cylinder	2411.53	9429.19	25.58
Hydraulic system	7722.69	30,425.88	25.38

### Discussion

According to the simulation results, the overall efficiency of the hydraulic system optimized by the optimization algorithm is improved by 3.49%. The efficiency of the knee joint hydraulic cylinder increases by 2.00%–2.60, while the efficiency of the hip joint hydraulic cylinder decreases by 2.33%–3.32. Since the knee joint consumes more energy, the overall efficiency of the hydraulic system is improved.

During the switching process of high-pressure and low-pressure power source, it is easy to generate the impact force due to the sudden change of pressure. The impact force increases with the increase of pressure difference between high-pressure and low-pressure power source. The impact force will reduce the stability during movement. Also, the instantaneous flow into the actuator will increase at the impact moment, which will increase the instantaneous power and energy consumption of the joint actuator. According to the comparison between the optimized and not optimized system, the variations of the parameters are shown in Table 6. In Table 6, “ + ” means that the parameters of the optimized system are larger.

**Table 6:** Increasing rate of the optimized system parameters.

	Hip joint			Knee joint		
	Peak power	Active work	Energy consumption	Peak power	Active work	Energy consumption
Left side	+ 45.31%	+ 29.94%	+ 39.80%	+ 22.45%	+ 0.89%	-6.39%
Right side	+ 49.33%	+ 21.97%	+ 33.64%	+ 23.56%	+ 3.43%	-6.14%

In order to verify the applicability of the algorithm, simulations on different gaits are carried out. The gaits make the robot walk in constant speed during the simulation. The system configuration and parameters are the same as mentioned in Section “System Configuration” and “Mathematical Model”. In the original system, the pressure range of the low-pressure accumulator is set as 10–12 MPa and the high-pressure accumulator is set as 19–21 MPa. The proposed algorithm is utilized to optimize the pressure range of the low-pressure accumulator. The simulation results show that the proposed algorithm can improve the overall energy efficiency of the hydraulic system from 2.88% to 3.93%. The detailed simulation process is omitted due to the limitation of the spaces. The simulation results are shown in Table 7.

**Table 7:** Increasing rate of the optimized system parameters.

Gait	Before optimization			After optimization		
	Active work/J	Energy/J	Efficiency/%	Active work/J	Energy/J	Efficiency/%
2 km/h walk	11,370.09	33,206.44	34.24	11,997.20	32,321.71	37.12
3 km/h walk	15,448.64	37,453.00	41.25	14,786.33	32,802.61	45.08
4 km/h walk	20,732.89	46,325.72	44.75	22,443.68	46,103.43	48.68

According to the analysis of the above results, the power unit pressure optimization algorithm has a better energy saving effect on the knee actuator. The reason is that the output force of the knee joint actuator is smaller than that of the hip joint without considering the impact load due to the different joint torque. Therefore, appropriately reducing the low-pressure oil supply pressure of the power unit is conducive for the knee joint actuator to the full use of the low-pressure circuit. This will contribute to reducing the throttling loss and improving the efficiency of the knee joint actuator.

On the other hand, high speed on/off valve is adopted to improve the pressure switching speed of the actuator, which will contribute to improving the response speed and reducing the pressure loss during switching. However, the simulation results show that the switching process will produce large pressure fluctuations. The pressure fluctuations adversely affect stability and increase energy consumption. In order to analyze the influence of the high-pressure and low-pressure switching process on the system energy consumption, the simulation model is further modified in this paper. The two-position two-way proportional valve is used to realize the pressure switching. Integrating element and saturation element are added into the switching signal to make the pressure of the servo valve port P rise or fall along a slop signal. The switching time is set as 0.1 s. In this model, the same movement gait is simulated and analyzed. The simulation results show that this method can reduce the impact force caused by pressure switching to a certain extent. But because of the delay of switching, the hydraulic system consumes more high-pressure power source to supply oil, which leads to the significant increase of energy consumption. Therefore, the selection of hydraulic system parameters needs to balance and optimize the contradiction between switching stability and energy consumption. This will be studied in the future work.

Additionally, the fluid flow required by the hydraulic actuator during the walking process of the biped robot varies widely. With such flow characteristics, accumulator pressure also presents strong fluctuation. The response characteristics of hydraulic pump and electric motor make it hard for the pump output flowrate to accurately equal to the consumed flow rate all the time. The pump can only provide an average flow rate. When the robot moves steadily with constant walking speed, the amount of oil consumed in each step is basically the same. In this case, the optimization algorithm can maintain the pressure variation range of the accumulator within the set value. The hydraulic system can make full use of the energy stored in the accumulator to achieve a better energy saving effect. However, when the robot’s walking speed changes, the average flow consumed by the actuator changes. The accumulator is prone to be under-charged, over-charged, or unable to reach the minimum set pressure, which makes the energy stored in the accumulator can not be fully used and the system energy consumption increases. So, the proposed optimization algorithm is more suitable for constant speed walking.

Finally, in order to verify the robustness of the algorithm, multiple calculations are carried out with the same gait. Due to the randomness of the Genetic Algorithm, the calculation results have fluctuation. The fluctuation range is no more than 0.1 MPa, which has no obvious influence on the energy efficiency of the hydraulic system.

## Conclusion

In this paper, an electric-hydraulic hybrid drive system is designed for the biped robot platform of Zhejiang Lab. The optimization algorithm of hydraulic power source pressure based on the genetic algorithm is proposed. Firstly, the model of the hydraulic system and mechanical structure is established. From the simulation analysis of the mechanical structure model, the motion and load characteristics of each joint under a specific action are obtained. Then, a value function reflecting the energy consumption of the hydraulic system is proposed. The value function calculates the energy consumption of the hydraulic system when a specific action is completed according to the pressure setting range of the low-pressure accumulator. A penalty function is introduced to control the switching time of the actuator between high and low pressure. Finally, genetic algorithm is used to calculate the low-pressure accumulator pressure setting range which makes the value function reach the minimum value.

According to the simulation analysis, the efficiency of the optimized hydraulic system is 3.49% higher than that of the unoptimized one. However, since the pressure difference between the high-pressure and low-pressure power sources is larger, it is easy to generate the impact force due to the sudden change of pressure. In order to further reduce the energy consumption of the hydraulic system, parameter matching of the hip and knee actuators should be carried out by combining the robot’s geometric scale, locomotive gait and the force. Also, the selection of hydraulic system parameters needs to balance and optimize the contradiction between switching stability and energy consumption.

## Data Availability

The data that support the findings of this study are available from the corresponding author, [P.Y.Z.], upon reasonable request.
